# An eHealth Diary and Symptom-Tracking Tool Combined With Person-Centered Care for Improving Self-Efficacy After a Diagnosis of Acute Coronary Syndrome: A Substudy of a Randomized Controlled Trial

**DOI:** 10.2196/jmir.4890

**Published:** 2016-02-23

**Authors:** Axel Wolf, Andreas Fors, Kerstin Ulin, Jörgen Thorn, Karl Swedberg, Inger Ekman

**Affiliations:** ^1^Institute of Health and Care SciencesSahlgrenska AcademyUniversity of GothenburgGothenburgSweden; ^2^Centre for Person-Centred CareUniversity of GothenburgGothenburgSweden; ^3^Närhälsan Research and Development, Primary Health Care, Region Västra GötalandGothenburgSweden; ^4^Department of Public Health and Community Medicine/Primary Health CareSahlgrenska AcademyUniversity of GothenburgGothenburgSweden; ^5^Närhälsan Primary Health CareRegion Västra GötalandGothenburgSweden; ^6^Department of Molecular and Clinical MedicineSahlgrenska AcademyUniversity of GothenburgGothenburgSweden; ^7^National Heart and Lung InstituteImperial CollegeLondonUnited Kingdom

**Keywords:** person-centred care: telemedicine, mobile health, eHealth, patient-centered care, self-efficacy, acute coronary syndrome

## Abstract

**Background:**

Patients with cardiovascular diseases managed by a person-centered care (PCC) approach have been observed to have better treatment outcomes and satisfaction than with traditional care. eHealth may facilitate the often slow transition to more person-centered health care by increasing patients’ beliefs in their own capacities (self-efficacy) to manage their care trajectory. eHealth is being increasingly used, but most studies continue to focus on health care professionals’ logic of care. Knowledge is lacking regarding the effects of an eHealth tool on self-efficacy when combined with PCC for patients with chronic heart diseases.

**Objective:**

The objective of our study was to investigate the effect of an eHealth diary and symptom-tracking tool in combination with PCC for patients with acute coronary syndrome (ACS).

**Methods:**

This was a substudy of a randomized controlled trial investigating the effects of PCC in patients hospitalized with ACS. In total, 199 patients with ACS aged <75 years were randomly assigned to a PCC intervention (n=94) or standard treatment (control group, n=105) and were followed up for 6 months. Patients in the intervention arm could choose to use a Web-based or mobile-based eHealth tool, or both, for at least 2 months after hospital discharge. The primary end point was a composite score of changes in general self-efficacy, return to work or prior activity level, and rehospitalization or death 6 months after discharge.

**Results:**

Of the 94 patients in the intervention arm, 37 (39%) used the eHealth tool at least once after the index hospitalization. Most of these (24/37, 65%) used the mobile app and not the Web-based app as the primary source of daily self-rating input. Patients used the eHealth tool a mean of 38 times during the first 8 weeks (range 1–118, SD 33) and 64 times over a 6-month period (range 1–597, SD 104). Patients who used the eHealth tool in combination with the PCC intervention had a 4-fold improvement in the primary end point compared with the control group (odds ratio 4.0, 95% CI 1.5–10.5; *P*=.005). This improvement was driven by a significant increase in general self-efficacy compared with the control group (*P*=.011). Patients in the PCC group who did not use the eHealth tool (n=57) showed a nonsignificant composite score improvement compared with those in the control group (n=105) (odds ratio 2.0, 95% CI 0.8–5.2; *P*=.14).

**Conclusions:**

We found a significant effect on improved general self-efficacy and the composite score for patients using an eHealth diary and symptom-tracking tool in combination with PCC compared with traditional care.

**Trial Registration:**

Swedish registry, Researchweb.org, ID NR 65 791.

## Introduction

Acute coronary syndrome (ACS) is an acute manifestation of coronary heart disease that includes myocardial infarction and unstable angina pectoris. In patients with ACS, eHealth studies have shown positive health-related outcomes [1-4]. “eHealth” is a relatively recent term for health care practice, which encompasses a variety of actions referring to health services and information delivered or enhanced through the Internet and related technologies [5,6]. As such, eHealth is an umbrella concept comprising all sorts of communication and information technology aimed at supporting and facilitating patients’ perception of well-being [7].

In contrast to eHealth, remote monitoring considers monitoring a disease from an objective perspective and implies 1-way communication between health care professionals and patients [8]. Such objective systems may limit the patient’s ability to participate in treatment decisions and to take full responsibility for their illness, which are essential elements in person-centered care (PCC). A PCC approach focuses on the patient as a person rather than on the disease alone, and implies that the patient has self-capacities that are valuable resources in an active partnership between the patient and health care professionals [9]. Increasing evidence suggests that patients with a diagnosis of cardiovascular disease who receive PCC, including active involvement in their care, shared decision making, and a structured follow-up, have better outcomes. Such improved outcomes include reduced uncertainty in illness, improved activity in daily living, a shorter hospital stay, and reduced costs of health care when compared with conventional care [10-12]. A central concept in PCC is self-efficacy [13], which is based on a person’s belief and confidence in achieving a certain task, rather than the actual execution and outcome of the task [14]. Higher levels of self-efficacy are associated with improved concordance between health care professionals and patients regarding prescribed treatment and increased physical activity for patients with congestive heart failure [15]. Findings of a recent review, focusing on chronic care management and eHealth, implied that most eHealth interventions are designed for 1-way communication and are driven by the logic of the health care professional rather than the patient’s [16]. Another review, analyzing over 350 studies within the area of eHealth and chronic disease management [17], showed that the majority of eHealth interventions studied were monitoring signs, and very few of those studies (n=4), in fact none within the cardiovascular field, had self-rated symptom reporting, hence sidestepping the patients’ experience of their illness and symptoms.

Consequently, there is a lack of knowledge about whether such solutions can be used in a PCC approach to strengthen a patient’s self-efficacy. Therefore, this study aimed to investigate the effect of a Web- and mobile-based eHealth diary and symptom-tracking tool (henceforth eHealth tool) combined with a PCC intervention in patients hospitalized for an ACS event.

## Methods

### Study Design and Setting

This study was part of a randomized intervention study: Person-centered Care after Acute Coronary Syndrome (PACS study, Swedish registry, Researchweb.org, ID NR 65 791) [18]. The PACS study evaluated the effects of a PCC intervention in patients with ACS throughout 3 health care levels (hospital, outpatient clinics, and primary care) compared with usual care alone. A detailed description of the study methods and findings has been reported previously [18]. In summary, patients were eligible for study inclusion if they were younger than 75 years, admitted for suspected ACS, and subsequently diagnosed with either myocardial infarction or unstable angina pectoris. Patients were included at 2 hospital sites within a university hospital setting in the western part of Sweden. Patients were excluded at admission if they met at least one of the following exclusion criteria: aged ≥75 years; not willing to participate; currently listed at a private primary care center or at a primary care center in another region; having no permanent address; being planned for heart surgery, such as coronary artery bypass grafting; having cognitive impairment; having known alcohol or drug abuse; having a survival expectancy of <1 year; or participating in a conflicting study. A total of 199 patients were randomly assigned in the main PACS study, with 105 patients in the control group and 94 patients in the intervention group.

For this substudy, all of the patients in the control group of the original PACS study were included and compared with those in the intervention group of PACS who chose an eHealth tool (eHealth group). The patients who were included in the eHealth group received the same structured PCC approach as described in the main PACS study [18] and were also given the choice to use the optional eHealth tool as a complement. Based on a structured PCC approach, every patient received the PCC intervention regardless of whether they chose the eHealth tool. Briefly, this approach builds upon the patients’ narrative used to identify their personal opportunities and barriers during cardiac rehabilitation after ACS. The condensed narrative, agreed on by the patient, physician, and registered nurse (PCC team), is documented in a PCC health plan. The PCC health plan includes the patient’s goals, expectations, and follow-up actions (date, time, and place). The focus is on each person’s resources and is the joint responsibility of both the health care professionals and the patients [9]. The PCC teams at each health care level (hospital, outpatient, and primary care) had access to the PCC health plan throughout this continuum of care, and discussed and revaluated or altered the PCC health plan with the patient if necessary [18].

The eHealth tool consisted of a mobile app and access to a webpage, and the patient had the option to use the webpage or the mobile app, or both. Patients who were enrolled in the control group were managed according to standard rehabilitation, which followed guideline-directed care that was compliant with Swedish standards. Patients in the control group answered questionnaires and instruments, similar to the eHealth group, at baseline, 4 weeks, 8 weeks, and 6 months.

### The eHealth Intervention

#### Mobile App

The mobile app consisted of 3 modules: (1) a self-rated fatigue scale, (2) a symptom trend graph, and (3) a built-in accelerometer within the phone to provide a daily average of the patient’s physical activity level (Table 1). Because fatigue is a common symptom after ACS [19], the self-rating scale was inspired by the Multidimensional Fatigue Inventory questionnaire by Smets et al [20]. The original Multidimensional Fatigue Inventory questionnaire is a validated, 20-item multidimensional fatigue questionnaire consisting of 5 dimensions: general fatigue, physical fatigue, activity, motivation, and mental fatigue. To minimize the number of items and still cover these dimensions, we enabled patients to self-rate their symptoms of physical and mental fatigue, as well as their motivation and activity levels (Table 2). The activity measurement within the mobile app automatically collected data throughout the entire day from the built-in accelerometer. The app calculated a mean daily level of energy expenditure, which was visualized for the patient on a symptom trend graph to be followed up and evaluated with registered nurses in the project if necessary. Patients also had the opportunity to show their trend graph to health care professionals during the follow-up period.

**Table 1:** Functional similarities and differences between the webpage and the mobile app eHealth interventions.

Webpage	Mobile app
Rating of fatigue	Rating of fatigue
Visual symptom trend graph over time	Visual symptom trend graph over time
Free-text diary function	Daily activity measurement using a built-in accelerometer
Chat function	
Personal links to relevant webpages	

**Table 2:** Patient self-rating of fatigue used in the webpage and mobile app eHealth interventions.

Dimension	Rating
Physical fatigue	I feel that I am in great condition
	I feel that I am in good condition
	I feel that I am in fair condition
	I feel that I am in poor condition
Mental fatigue	I have no problem concentrating
	I have to make an effort to concentrate
	I have to make a huge effort to keep concentrating
	I cannot concentrate at all
Motivation	I want to do a lot of things
	I only do the most necessary things
	I have no motivation to do anything
	I dread doing anything at all
Activity level	I feel very active
	I manage what needs to be done
	I get very little done
	I do nothing

#### Webpage

The webpage consisted of 5 modules: (1) self-rated symptoms of fatigue (same as on the mobile app described above), (2) a symptom trend graph, (3) a diary function for free-text entries to capture the everyday experience using the patient’s own words, (4) a chat function with other patients and registered nurses within the study, and (5) personal links to relevant webpages and the ability to upload documents (Table 1). The text diary was open for text input until midnight the same day. After this time, the patient could not revise the written text regarding that day. The webpage and the mobile app synchronized the data.

A registered nurse at the hospital asked all of the patients in the eHealth group if they were interested in using the eHealth tool. Patients had the opportunity to borrow a mobile phone with the eHealth app preinstalled or to download it for use on their own mobile phone. Users were registered with a username and password on the webpage, and the online webpage was connected to the mobile app. An introductory demonstration, which required the patient to test the eHealth tools, was provided by a registered nurse who was familiar with the study so that patients could start using the tools freely during their hospital stay. Additional training could be requested if needed. Patients also had access to a video demonstration online for further information. The patients themselves decided on the frequency and patterns of use of the eHealth tools. After 8 weeks, the registered nurse and physician at the primary care center asked patients whether they wanted to return (if borrowed) or continue to use the mobile phone. Access to the webpage had no time restriction.

### Instruments

We evaluated patient-reported scores on the General Self-Efficacy Scale (GSES) using the Swedish version [21] of the original GSES [22]. The GSES, a unidimensional scale and universal construct, is validated in several countries [23]. The Swedish validated version of the GSES has high internal consistency (alpha = .90) [21]. The GSES is a 10-item instrument that measures patients’ beliefs and confidence in accomplishing certain tasks, rather than the actual execution and outcome of these tasks. Each item is rated by the patient on a 4-point Likert scale, in which 1 = not at all true, 2 = hardly true, 3 = moderately true, and 4 = exactly true. Total scores ranging from 10 to 40 are calculated, with higher totals indicating higher levels of general self-efficacy.

Patients in the control and eHealth groups filled out the GSES instrument at baseline at the hospital, and at 4 weeks, 8 weeks, and 6 months.

### Primary End Point

The primary end point was a composite of changes in general self-efficacy, return to work or prior activity level, and rehospitalization or death. Each patient was classified as improved, deteriorated, or unchanged. An increase of 4.6 units in the GSES has been suggested to show the minimal clinical important difference for patients [24]. A patient was classified at 6 months as improved in the composite score as follows: self-efficacy had increased by ≥5 units and the patient was not readmitted for unscheduled cardiovascular reasons or death; and the patient had returned to work or previous physical activity level (improved from sedentary to moderate activities or better, or maintained or improved from moderate to demanding or strenuous activities) [25].

Those patients who had neither deteriorated nor improved were considered unchanged. Patients were dichotomized into improved versus deteriorated or unchanged status.

### Statistical Analyses

Patients in the PCC intervention group who had used the eHealth tool at least once after discharge were included into this substudy and compared with the control group. We used descriptive statistics, such as frequency, mean, median, range, and SD, to describe user patterns. Between-group differences were tested using Fisher exact test for dichotomous variables and the Mann-Whitney *U* test was used for continuous variables. Logistic regression was used to calculate the odds ratios (ORs) between groups, with a 95% CI. We analyzed the data using SPSS 22 (IBM Corporation) statistical software package.

### Ethics

The Regional Ethics Committee of the University of Gothenburg approved the study (DNr 275-11). The study adhered to the rules of the Declaration of Helsinki of ethical principles.

## Results

**Figure 1:**
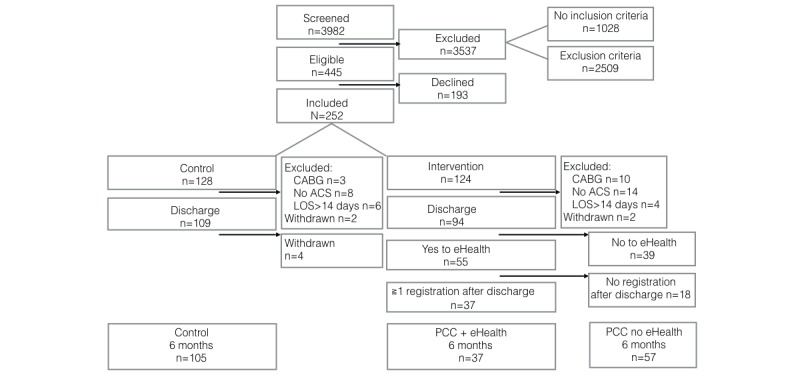
Study profile. ACS, acute coronary syndrome; CABG: coronary artery bypass graft; LOS: length of hospital stay; PCC: person-centered care.

Of the 94 patients in the intervention arm, 37 (39%) chose to use the eHealth tool (PCC + eHealth) and continued to use it at least once, even after discharge from the hospital. The remaining patients (PCC no eHealth, n=57) did not choose to use the eHealth tool (n=39) or did not use the eHealth tool after discharge (n=18) (Figure 1). The majority of patients were male, with a mean age of 60 years (SD 10). There were no significant differences in demographic characteristics, such as age, education, socioeconomic level, diagnosis, or general self-efficacy between patients in the different groups at baseline (Table 3). The majority of patients in the PCC + eHealth group (24/37, 65%) used the mobile app rather than the Web-based app as the primary source of daily self-rating input. Patients used the eHealth tool a mean of 38 times during the first 8 weeks (range 1–18, SD 33) and 64 times over a 6-month period (range 1–597, SD 104).

**Table 3:** General characteristics of the study population divided into control versus PCC^a^+ eHealth and PCC no eHealth.

Characteristic		Control (n=105)	PCC + eHealth (n=37)	PCC no eHealth (n=57)
Female, n (%)		32 (30.5)	7 (19)	16 (28.1)
Age in years, mean (SD)		61.3 (8.9)	59.8 (10.1)	60.9 (8.7)
**Education, n (%)**				
	None	1 (1.0)	1 (3)	0 (0)
	Compulsory	21 (20.0)	5 (14)	11 (19)
	Secondary school	28 (26.7)	7 (19)	16 (28)
	Vocational college	14 (13.3)	9 (24)	12 (21)
	University	41 (39.0)	15 (41)	18 (32)
Employed, n (%)		60 (57.1)	24 (65)	30 (53)
**Income, n (%)**				
	Low	13 (12.4)	5 (14)	10 (18)
	Lower-middle	20 (19.0)	4 (11)	9 (16)
	Upper-middle	30 (28.6)	18 (49)	17 (30)
	High	30 (28.6)	8 (22)	16 (28)
	Missing data	12 (11.4)	2 (5)	5 (9)
**Type of acute coronary syndrome, n** (%)				
	ST-elevation myocardial infarction	24 (22.9)	9 (24)	15 (26)
	Non-ST-elevation myocardial infarction	51 (48.6)	13 (35)	25 (44)
	Unstable angina	30 (28.5)	15 (41)	17 (30)
General self-efficacy, mean (SD)		30.3 (5.6)	28.8 (6)	30.0 (6)

^a^PCC: person-centered care.

A higher percentage of patients (11/37, 30%) in the PCC + eHealth group improved in the composite score than those in the control group (n=105) over a 6-month period (OR 4.0, 95% CI 1.5–10.5; *P*=.005) (Table 4). There was a significant increase in mean self-efficacy levels, as measured by the GSES, at 6 months in the PCC + eHealth group (n=37) compared with the control group (n=105) (*P*=.01). Patients in the PCC no eHealth group (n=57) showed a nonsignificant improvement in the composite score compared with those in the control group (n=105) (OR 2.0, 95% CI 0.8–5.2; *P*=.14). When comparing the PCC group without eHealth versus the PCC group + eHealth, the PCC + eHealth group (n=37) showed a nonsignificant improvement in the composite score compared with the PCC no eHealth group (n=57) (OR 2.0, 95% CI 0.7–5.3; *P*=.17). In both these comparisons, no significant differences were observed in mean self-efficacy levels at 6 months.

There were 6 events in the PCC + eHealth group (1 death, 5 readmissions), 12 events in the PCC group without eHealth (3 deaths, 9 readmissions), and 16 events in the control group (2 deaths, 14 readmissions). The proportion of patients who returned to work was similar between groups at 6 months (PCC + eHealth 30/34, 88%; PCC no eHealth 47/53, 89%; control 89/98, 91%).

**Table 4:** Primary end point: change in composite score dichotomized into improved versus unchanged or deteriorated condition in the control group compared with PCC^a^with or without an eHealth intervention.

Change in composite score at 6 months		Control (n=105)	PCC + eHealth (n=37)	PCC no eHealth (n=57)
Improved, n (%)		10 (9.5)	11 (30)	10 (18)
	*P* value vs control		.006	.21
Unchanged or deteriorated, n (%)		95 (90.5)	26 (70)	47 (83)

^a^PCC: person-centered care.

## Discussion

We found that patients who used the eHealth tool in combination with PCC had a 4 times higher improvement in the primary end point compared with those receiving usual care. However, fewer than half of the eligible patients used the eHealth tool after discharge, and they preferred the mobile app over the webpage. In comparison with the patients receiving PCC who did not choose or did not use the eHealth tool after the index hospitalization, improvement in the primary end point was less prominent.

### eHealth Tool Use Patterns

This study showed feasibility, to a limited extent, for use of an eHealth tool by patients after an ACS event because approximately 40% of the eligible patients used the eHealth intervention after discharge. Patients were offered use of the eHealth tool as a completely optional supplement without any reminders. ACS is an overwhelming event inducing several concerns during hospitalization [26]; therefore, the adherence rate for using the eHealth tool might have been increased if the patients had been asked again if they wanted to use an eHealth tool when their health status had been stabilized, that is, after hospital discharge. The findings from this study are congruent with a systematic review by Munro and coworkers [27] showing use of eHealth programs for targeting patients with different diagnoses of cardiovascular disease, ranging from 36% to 97%. Interestingly, a meta-analysis by Inglis and coworkers [28] in patients with congestive heart failure showed a slightly higher use of eHealth, ranging from 66% to 98%. Nevertheless, because of the design of the study, the use pattern may indicate the present uptake levels for these kinds of self-management tools in the clinical setting. Similar to our findings regarding the use pattern of a mobile app versus a computer-based app, a study with patients with myocardial infarction evaluating uptake of technology-assisted cardiac rehabilitation showed that patients used the mobile app more frequently than a computer-based tool [29]. In this study the eHealth tool had more functions on the Web than on the mobile app, which may have negatively affected the adherence rate.

Clark et al [2] suggested that the duration of the interventions could have a negative effect on eHealth use for patients with ACS because interventions shorter than 2 months involved more users than interventions lasting longer than 2 months. In this study, the average use of the eHealth tool increased during the study period, indicating that patients who used the eHealth app for 2 months or more also used it most often. This finding needs to be further examined in future studies.

### Self-Efficacy

Whereas we observed no difference between groups regarding death or rehospitalization, the primary end point was determined by an improvement in the patients’ self-efficacy level. Self-efficacy is a person’s belief in his or her own ability to execute the behavior required to achieve desired outcomes [14,30]. According to this theory, self-efficacy can be influenced and strengthened, which is probably relevant to patients with chronic conditions. Dickson et al [31] suggested that self-efficacy in patients with congestive heart failure is a dynamic, oscillating resource that is enhanced or diminished by the context and situation. Nevertheless, because health care professionals focus on the medical needs of hospitalized patients, they seldom systematically consider patients’ personal resources, independence, and preferences [9]. The essence of PCC is the partnership between the patient and health care professionals. This partnership is based on a shared knowledge and mutual agreement about living with illness (patient) and generic knowledge about the disease (health care professionals). The way in which patients view their illness is as important as the disease itself and an essential factor for improving health outcomes and returning to professional work after an ACS event [32]. The eHealth tool in this study was developed to be used as a self-care device. Increased knowledge about oneself in relation to the illness and disease in question could be an important step in strengthening patients’ role as an expert about their everyday life and an active partner in the interaction with health care professionals.

The eHealth tool also made it possible for patients and health care professionals to develop a partnership through their communication via the chat function on the webpage and by patients presenting their trend graphs during follow-up visits. Since this is a complex intervention it is difficult to differentiate which component of the PCC intervention contributed most to the improvement in general self-efficacy. This study suggested that an eHealth tool in addition to a PCC intervention was associated with even higher improvement levels in the composite score in this selected group of patients than in the control group. The effect was driven by improved general self-efficacy, which suggests that an eHealth tool added to a PCC intervention can improve patients’ beliefs in their ability to successfully respond to challenges across a wide range of situations. This in turn was shown to contribute to improved disease management and clinical outcomes, such as health status and health care utilization [33]. The potential of digital technologies to become disruptive innovations in traditional power structures such as health care lies not only in making processes more transparent and easily accessible for the end user. We believe that the disruptive force also could change health care providers’ perception of the patients’ own view of their capacities to manage different situations. Nevertheless, research shows that most eHealth studies in the field of chronic care management take a professionally driven approach to monitoring signs [16,17], where professionals try to activate patients by pushing content and information that the professionals believe is of importance for the patient. Instead, we propose a more active patient role where the patients themselves seek information and create knowledge that is of importance to them in order to develop a productive interaction that fits the need of both the patients and the health care professionals. Hence, more studies need to investigate the potential in changing the perception of the patient’s role as a passive provider of data to an active cocreator of knowledge.

While 94 patients were included in the PCC intervention arm, only 37 (39%) chose the eHealth tool. Nevertheless, 11 patients (30% of the active users, or 12% of the total intervention group) improved even more in the primary end point when they complemented PCC with the eHealth tool in comparison with PCC alone. A comparison of this study outcome with that in the original paper published by Fors et al [18] indicates that the eHealth tool could have an augmenting effect on PCC. The primary end point was a composite score, combining patient experiences with clinical outcomes, which have been shown to be sensitive in differentiating treatment outcomes [34]. We defined improvement very restrictively as requiring no rehospitalization or death in combination with improvement on the GSES by ≥5 units (equivalent to almost 1 SD; in general, 0.5 SD is considered of clinical importance [35]). We also included return to work or previous activity level as a measure of improvement. Our specific definition of improvement might explain why only a minority benefitted from the intervention. For the effects of the intervention to be reflected as a hard outcome, a larger sample and longer follow-up period are needed. In general, there is a low power to evaluate subgroup analyses in clinical trials. Thus, the effects on clinical outcomes need to be studied in larger studies [36].

eHealth was still not considered as a viable support tool by the majority of patients in this study. Qualitative studies in telecare suggest that patients with congestive heart failure emphasize the value of the relationship with their health care professional [37]. Additionally, the patient’s primary concern with telecare is that it should not disrupt or compensate for ordinary face-to-face services [38]. Patients’ fear that eHealth is a replacement for face-to-face meetings could affect the decision not to choose an eHealth tool. Therefore, future studies should examine how the interaction and communication aimed at improving self-efficacy in people with long-term diseases can be developed through eHealth solutions or other means. In addition, studies need to investigate how patients and their relatives could better understand their own role in PCC. We believe that the interactive communication as manifested *by* the partnership in PCC can provide substantial *support* for eHealth and differentiate this technology from telemedicine. However, this needs to be confirmed in future studies.

### Limitations

This study has several limitations. Results should be interpreted based on the limitation that this was a substudy. Only approximately 40% of the patients included in the intervention agreed to participate in this study, which used an eHealth tool at least once after discharge. This group could consist of the most motivated individuals. Additionally, comparing these patients with the entire control group was a limitation of this study. However, there were no significant differences at baseline in demographic variables between the control and intervention groups. Despite this limitation, our study suggests that, for a selected group of people, this type of eHealth tool adds value in combination with PCC. Finally, another limitation is that we did not know whether the patients actively used the eHealth solution as part of the follow-up visits at the outpatient clinic or in primary care. While patients who used the eHealth tool had significantly higher general self-efficacy levels compared with the control group, the effect of using eHealth tools on shared decision making in a PCC setting still needs to be investigated. More studies, also using a qualitative approach, need to evaluate the potential of the intervention in terms of understanding the tool and patients’ own role in PCC and among a broader study population.

### Conclusion

An eHealth diary and symptom-tracking tool in combination with a structured PCC intervention is associated with improved combined scores, comprising self-efficacy, return to work or prior activity level, rehospitalization, and death, in a selected group of patients with ACS compared with usual care. Future research should address the effects and efficiency of an eHealth tool throughout PCC interventions compared with traditional care.

## Abbreviations

ACS: acute coronary syndrome
GSES: General Self-Efficacy Scale
OR: odds ratio
PACS: Person-centered Care after Acute Coronary Syndrome
PCC: person-centered care
